# PARP-2 and PARP-3 are selectively activated by 5′ phosphorylated DNA breaks through an allosteric regulatory mechanism shared with PARP-1

**DOI:** 10.1093/nar/gku474

**Published:** 2014-06-07

**Authors:** Marie-France Langelier, Amanda A. Riccio, John M. Pascal

**Affiliations:** Department of Biochemistry & Molecular Biology, Kimmel Cancer Center, Thomas Jefferson University, Philadelphia, PA 19107, USA

## Abstract

PARP-1, PARP-2 and PARP-3 are DNA-dependent PARPs that localize to DNA damage, synthesize poly(ADP-ribose) (PAR) covalently attached to target proteins including themselves, and thereby recruit repair factors to DNA breaks to increase repair efficiency. PARP-1, PARP-2 and PARP-3 have in common two C-terminal domains—Trp-Gly-Arg (WGR) and catalytic (CAT). In contrast, the N-terminal region (NTR) of PARP-1 is over 500 residues and includes four regulatory domains, whereas PARP-2 and PARP-3 have smaller NTRs (70 and 40 residues, respectively) of unknown structural composition and function. Here, we show that PARP-2 and PARP-3 are preferentially activated by DNA breaks harboring a 5′ phosphate (5′P), suggesting selective activation in response to specific DNA repair intermediates, in particular structures that are competent for DNA ligation. In contrast to PARP-1, the NTRs of PARP-2 and PARP-3 are not strictly required for DNA binding or for DNA-dependent activation. Rather, the WGR domain is the central regulatory domain of PARP-2 and PARP-3. Finally, PARP-1, PARP-2 and PARP-3 share an allosteric regulatory mechanism of DNA-dependent catalytic activation through a local destabilization of the CAT. Collectively, our study provides new insights into the specialization of the DNA-dependent PARPs and their specific roles in DNA repair pathways.

## INTRODUCTION

The PARP superfamily is composed of 17 members, which all share a conserved ADP-ribosyl transferase (ART) fold, and regulate a multitude of cellular processes (1–3). The founding and most studied member, PARP-1, was named for its ability to produce polymers of ADP-ribose (PAR) using NAD^+^ as a substrate. PARP-1 synthesizes PAR attached to proteins, including itself, as a post-translational modification that regulates the function of modified proteins. Among the PARP family members, only a subset is predicted to have the ability to produce PAR (PARP-1 to PARP-5a and PARP-5b) while two are inactive enzymes (PARP-9 and PARP-13) and the remaining members are able to produce a mono-ADP-ribose modification (4).

PARP-1, PARP-2 and PARP-3 are DNA-dependent enzymes that are catalytically activated upon binding to DNA damage (1,5,6) and play important roles in the repair of DNA strand breaks (7). In cells, PARP-1, PARP-2 and PARP-3 recruit to sites of DNA damage induced by laser microirradiation or site-specific nucleases (8–10). PARP-1 is involved in the repair of both single-strand and double-strand breaks (SSBs and DSBs) and influences multiple repair pathways, including base excision repair (BER), homologous recombination (HR), alternative non-homologous end-joining (a-NHEJ) and nucleotide excision repair (NER). Less is know about PARP-2 and PARP-3 involvement in DNA repair. PARP-2 depletion leads to sensitivity to ionizing radiation and alkylating agents (11,12), consistent with a role in SSB repair. PARP-2^−/−^ cells exhibit slower kinetics of re-joining DNA strand breaks (11). PARP-2 interacts with various players of the BER pathway, including XRCC1 and DNA ligase III (11). Additionally, PARP-2 is proposed to function in HR in a way similar to PARP-1 (13). PARP-3 plays a role in DSB repair by promoting the recruitment of aprataxin-like factor (APLF) to sites of damage (6). PARP-3 and APLF are expected to increase the efficiency of DSB repair by increasing recruitment/retention of the XRCC4/DNA LigIV complex at DNA breaks (6).

**Figure 1.**
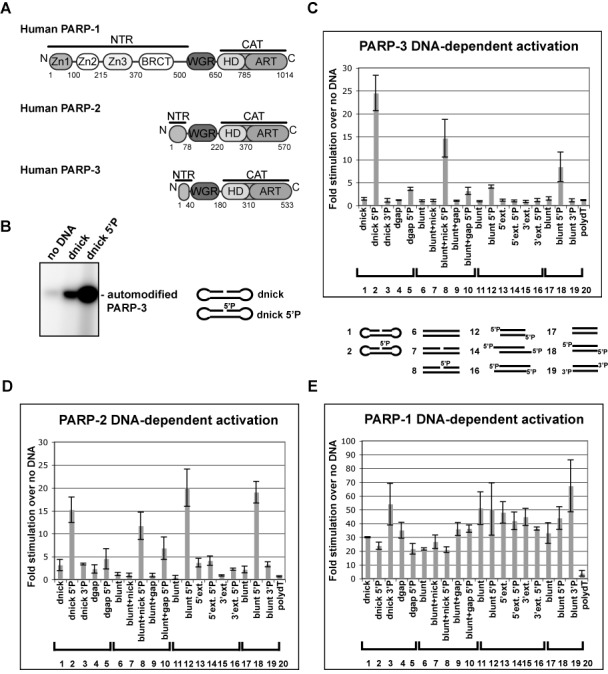
PARP-2 and PARP-3 are selectively activated by 5′ phosphorylated DNA breaks. (**A**) Domain architecture of PARP-1, PARP-2 and PARP-3. The WGR and CAT domains are conserved, while the N-terminal regions (NTRs) are distinct. (**B**) Radioactive assay showing PARP-3 automodification activity in the absence or presence of DNA. Protein (1.5 μM) and DNA (2.4 μM) were incubated for 1 h in the presence of 25 μM NAD^+^ (5 μM ^32^P-NAD^+^, 20 μM NAD^+^). dnick and dnick 5′P are dumbbell templates containing a region of 19 base pairs (bp) with a 5′ phosphorylated or non-phosphorylated nick after bp 10 and 4 nucleotide turns at the extremities. (**C**) Colorimetric assay showing stimulation of PARP-3 DNA-dependent activity by a panel of DNA structures (60 nM protein, 480 nM DNA, 1 h time point). Stimulation is calculated as the ratio of activity measured in the presence versus absence of DNA. The average of three independent experiments is shown with associated standard deviations. Templates 1–5 are dumbbells derived from the dnick template described in (B). The dnick 3′P has a phosphate on the 3′ terminus. The dgap templates have a one-nucleotide gap instead of the nick. Templates 6–10 are 47 bp duplexes (blunt), with either a nick after bp 24 (blunt + nick) or a one-nucleotide gap (blunt + gap). Templates 11–16 are based on a 26-bp palindromes (blunt), with a two-nucleotide 5′ or 3′ extension (5′ext. or 3′ext.). Templates 17–19 are 28 duplexes template with either a 5′ OH (blunt), a 5′P (blunt 5′P) or a 3′P (blunt 3′P). Template 20 is a single-stranded DNA containing 16 dTs. See Supplementary Figure S1 for more details on DNA templates. (**D** and **E**) Same as (C) for PARP-2 and PARP-1 with a 15-min time point.

PARP-1, PARP-2 and PARP-3 share a conserved C-terminal region, but differ greatly in their N-terminal regions (NTRs) (Figure 1A). PARP-1 is 116 kDa and composed of six independently folded domains. Two N-terminal zinc fingers, Zn1 and Zn2, are involved in binding to DNA breaks (14–17), which stimulates PARP-1 activity up to 500-fold (1). A third zinc-binding domain, Zn3, plays a role in binding to DNA, transmitting the DNA binding signal to the CAT, and compacting chromatin structure (14,18–20). The automodification domain (AD) contains a BRCT fold and several of the residues that are targeted for automodification. The WGR domain participates in binding DNA near the 5′ terminus and mediates domain–domain contacts essential for DNA-dependent activity (20). The CAT domain, which is composed of two subdomains (helical subdomain—HD, and ART), is responsible for binding the substrate NAD^+^ and for the synthesis of PAR. In PARP-1, the Zn1, Zn3, WGR and CAT domains have been shown to be essential for DNA-dependent activity on DSB (14,18,21,22), while Zn2 plays an important role in activation by SSBs (23). Recently, the crystal structure of a complex of all the essential domains of PARP-1 (Zn1, Zn3, WGR-CAT) bound to a DNA break has shed light on how PARP-1 binding to DNA results in stimulation of its catalytic activity (20). Zn1, Zn3 and WGR domains assemble on the DNA break and form a network of interdomain contacts that ultimately lead to destabilization of the HD and increased activity of the ART (20,24).

PARP-2 (65 kDa) and PARP-3 (63 kDa) both have WGR and CAT domains similar to PARP-1. In contrast to PARP-1, the NTRs of PARP-2 and PARP-3 are much shorter (78 and 40 residues, respectively) (Figure 1A), and no structural information is available for these regions. It has been proposed that the NTR of PARP-2 is involved in DNA binding, due to the presence of basic residues in this region (5), and because sequence analysis has suggested that certain PARP-2 homologs contain a DNA-binding SAF-A/B, Acinus and PIAS (SAP) domain found in various nuclear proteins (25). Additionally, an RNA binding activity has been recently proposed for the NTR of PARP-2 (26). However, further experimental data is needed to understand the role of PARP-2 and PARP-3 NTRs in DNA binding and activation. Moreover, little is known about the role of PARP-2 and PARP-3 WGR domains in DNA-dependent activation and the mechanism of transmitting the DNA binding signal to the CAT domain leading to PARP-2 and PARP-3 activation.

PARP-1 is activated by several types of damaged DNA structures including DSBs, SSBs, overhangs, hairpins and cruciforms (14–17,27). PARP-2 and PARP-3 have been less extensively studied in terms of their DNA structure preferences for activation. PARP-2 was shown to bind to flap and gap containing DNA templates (28). PARP-3 is activated by DNAs containing DSBs (6), consistent with its role in DSB repair. Further details of the PARP-2 and PARP-3 DNA-dependent mechanism of activation are needed to understand how these PARPs can play specific roles in repair pathways as part of the cellular response to DNA damage. In the present study, we show that PARP-2 and PARP-3 exhibit a strong preferential activation by 5′ phosphorylated DNA breaks. 5′ phosphorylated nicks are particularly efficient activators of PARP-2 and PARP-3, suggesting a role for PARP-2 and PARP-3 in the process of responding to and correcting this particular type of DNA repair intermediate that precedes the final ligation step of DNA damage repair.

We have also investigated the contribution of the NTRs of PARP-2 and PARP-3 to DNA binding affinity and catalytic activation. Our results show that the NTRs of PARP-2 and PARP-3 play a less critical role in overall DNA binding affinity compared to that of the PARP-1 NTR. We also show that PARP-2 and PARP-3 NTRs are not strictly required for DNA-dependent activity, as opposed to the essential requirement of the PARP-1 NTR. Similar to PARP-1, the WGR domains of PARP-2 and PARP-3 play critical roles in DNA-dependent activation, making contacts with DNA and forming conserved interactions with the HD that are essential to the regulation of catalytic activity. Finally, we show that destabilization of the HD upon DNA binding is a common allosteric regulatory mechanism shared among PARP-1, PARP-2 and PARP-3, and 5′ phosphorylated DNA breaks specifically activate the allosteric regulatory mechanism of PARP-2 and PARP-3.

## MATERIALS AND METHODS

### Gene cloning and mutagenesis

The cDNA coding for human PARP-2 isoform 2 (NP_001036083) was provided by Dr. G. Poirier. The PARP-2 cDNA was cloned into the NdeI/XhoI restriction sites of the pET28b (Novagen) expression vector with an N-terminal hexahistidine tag. Human PARP-3 isoform b was expressed from a pDEST17 expression vector (hexahistidine tag) provided by Dr. I. Ahel. Gene mutations and truncations were performed using the QuikChange Protocol (Stratagene) and verified by automated sequencing (Kimmel Cancer Center).

### Protein purification

PARP-1 was purified as described previously (21,29). PARP-2 wild-type (WT) and mutant proteins were expressed in *Escherichia coli* strain BL21(DE3), and culture medium was supplemented with 10 mM benzamide in some cases (proteins in Figure 5A). PARP-2 was purified essentially as described for PARP-1 (29) with some modifications. In particular, Ni^2+^-column wash buffers were supplemented with 0.1% NP-40. PARP-2 proteins were eluted from the Ni^2+^-column in 20 mM HEPES (N-(2-Hydroxyethyl)piperazine-N′-2-ethanesulfonic acid TCEP, Tris(2-carboxyethyl)phosphine) pH 8.0, 500 mM NaCl, 0.1 mM TCEP, and 400 mM imidazole, then diluted to 425 mM NaCl (PARP-2 WT and mutants) or 300 mM NaCl (ΔNTR-PARP-2) prior to loading onto a 5 ml HP heparin column (GE Healthcare). Heparin fractions were dialyzed in the following buffer: 20 mM HEPES pH 8.0, 150 mM NaCl, 0.1 mM TCEP and 0.1 mM EDTA (ethylenediaminetetraacetic acid). ΔNTR-PARP-2 was purified over a S200 Sephacryl size exclusion column in the same buffer. PARP-3 was expressed in Rosetta^2^ (DE3) cells (Stratagene) and purified essentially as described for PARP-1 (21). After Ni^2+^-column elution, PARP-3 WT and mutant proteins were diluted to 50 mM NaCl prior to loading onto a 5-ml HP heparin column. Heparin fractions were further purified over a S200 Sephacryl size exclusion column in 20 mM HEPES pH 8.0, 150 mM NaCl, 0.1 mM TCEP and 0.1 mM EDTA.

**Figure 2.**
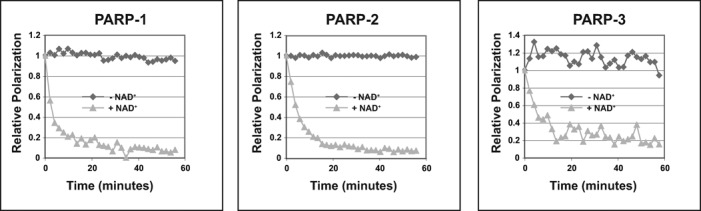
PARP-1, PARP-2 and PARP-3 release from DNA upon production of PAR. Fluorescence polarization release assay. Saturating amount of proteins were incubated with a mixture of unlabeled and labeled DNA probe and incubated for 30 min at RT. PARP-1: 200 nM protein, 100 nM DNA (28-bp duplex 5′P, template 18). PARP-2: 2.5 μM protein, 1.25 μM DNA (28-bp duplex 5′P, template 18). PARP-3: 4.0 μM protein and 1 μM DNA (47-bp duplex with 5′P nick, template 8). 1 mM NAD^+^ was added to start the ADP-ribosylation reaction and fluorescence polarization was measured over a time course. Relative polarization represents the ratio of the polarization measured at time × over the polarization measured at time zero, which was set to one. Representative curves of three replicates are shown.

**Figure 3.**
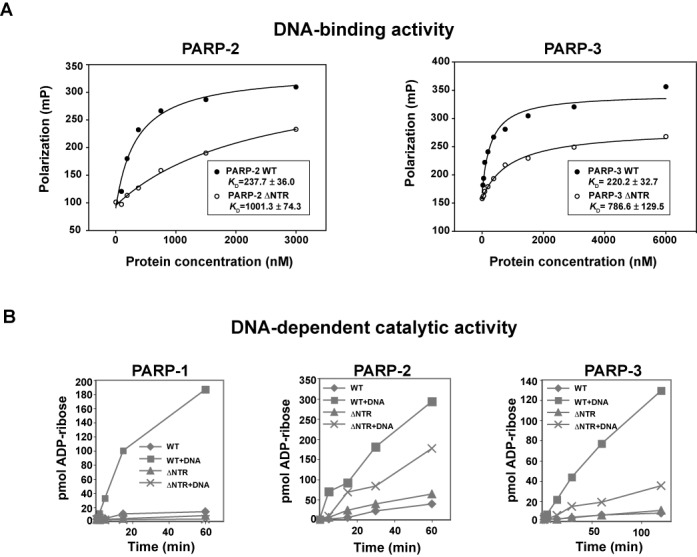
The NTRs of PARP-2 and PARP-3 are not strictly required for DNA binding and activation. (**A**) Fluorescence polarization DNA binding experiment for PARP-2 WT, PARP-3 WT and their respective NTR deletions using a fluorescently labeled 5′ phosphorylated DNA probe (5 nM). PARP-2 assay was performed using the 28-bp duplex 5′P (template 18). PARP-3 assay was performed using the 47-bp duplex with 5′P nick (template 8). The *K*_D_ is an average of three independent experiments with associated standard deviation. (**B**) Colorimetric assay showing the activity of ΔNTR-PARP-1, ΔNTR-PARP-2 and ΔNTR-PARP-3 compared to WT using 60 nM protein and 480 nM DNA. PARP-2 assay was performed using the 28-bp duplex 5′P (template 18). PARP-1 and PARP-3 assays used the dnick 5′P template (template 2). Representative curves of the three replicates are shown.

**Figure 4.**
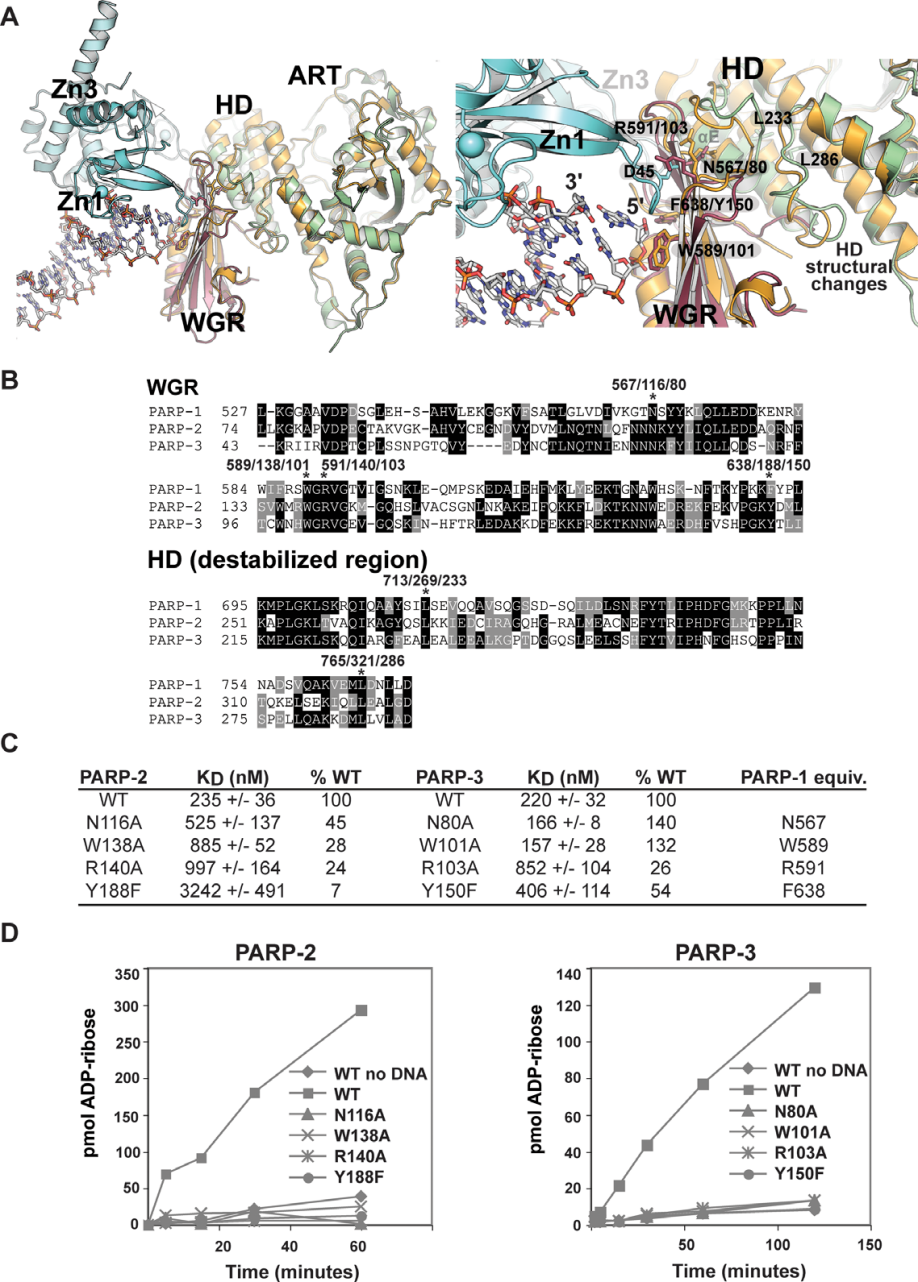
Structure-guided mutagenesis of the PARP-2 and PARP-3 WGR domains. (**A**) Structural alignment between the NMR structure of the PARP-3 WGR domain (PDB: 2EOC; magenta), a crystal structure of the PARP-3 CAT (HD/ART) (PDB: 3C49; green) and the PARP-1/DNA crystal structure (PDB: 4DQY; Zn1 and Zn3 in teal, WGR-CAT in orange). (**B**) Sequence alignment of human PARP-1, PARP-2 and PARP-3 WGR domain, and a region of the HD. Residues targeted for mutagenesis are marked with an asterisk. (**C**) The DNA binding affinity of PARP-2 and PARP-3 WGR mutants was determined by fluorescence polarization. The *K*_D_ reported represents an average of three independent experiments with associated standard deviation. Examples of binding curves are shown in Supplementary Figure S4. The PARP-2 assay used the fluorescently labeled 28-bp duplex 5′P (template 18, 5 nM). The PARP-3 assay used the fluorescently labeled 47-bp duplex with 5′P nick (template 8, 5 nM). (**D**) Colorimetric assay showing the activity of PARP-2 and PARP-3 WGR mutants using 60 nM protein, 480 nM DNA (PARP-2: 28-bp duplex 5′P, template 18; PARP-3: dnick 5′P, template 2). Representative curves of the three replicates are shown.

**Figure 5.**
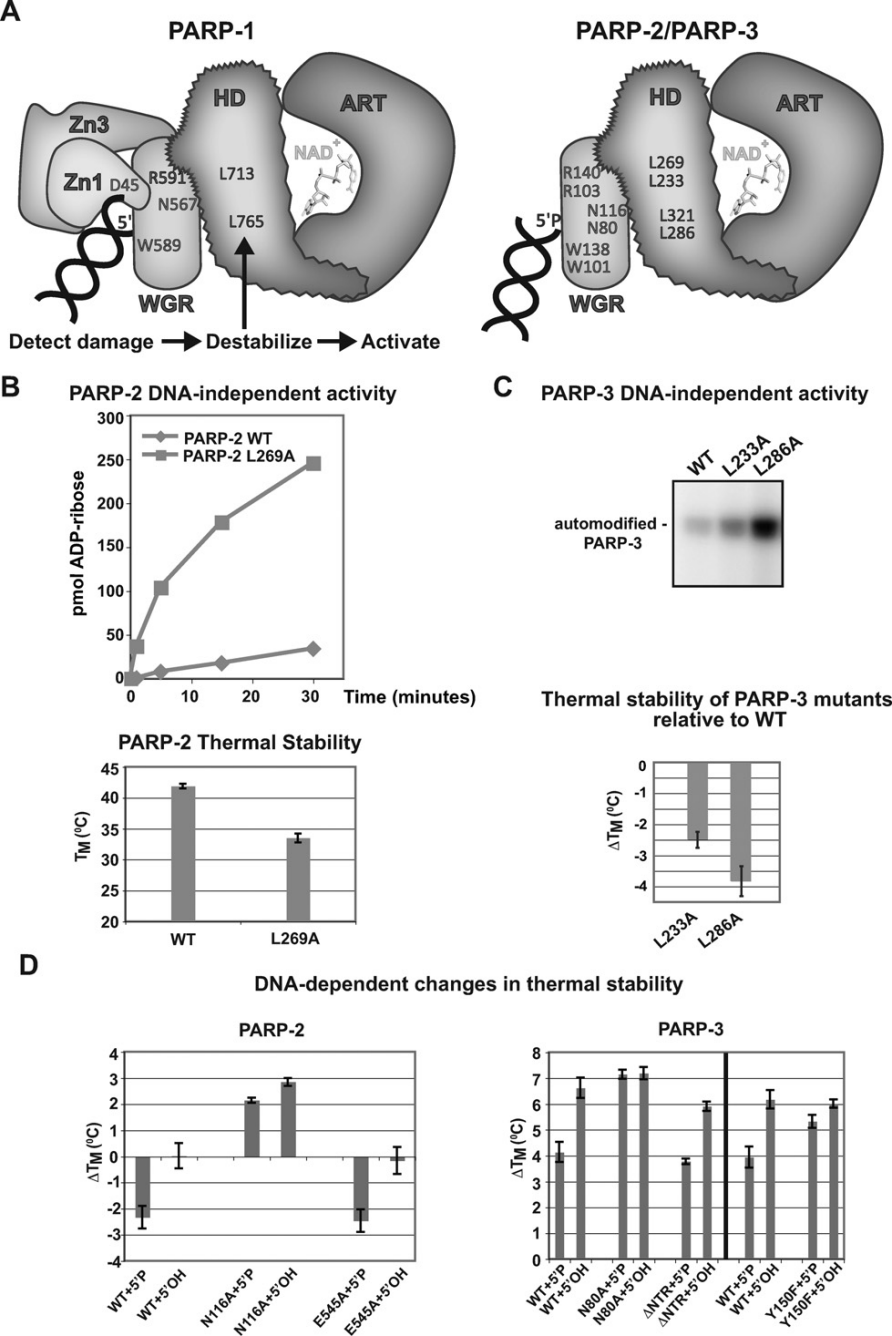
PARP-2 and PARP-3 share with PARP-1 a conserved mechanism of activation through HD destabilization. (**A**) Model for PARP-1, PARP-2 and PARP-3 DNA-dependent allosteric mechanism of activation. Left, PARP-1 regulatory domains (Zn1, Zn3, WGR) collapse onto DNA and form interdomain contacts that create destabilizing changes in the HD of the CAT and lead to ART activation. Right, our results suggest that the same mechanism of activation exists for PARP-2 and PARP-3; however, the WGR is their primary regulatory domain. Mutation of specific residues in the HD hydrophobic core mimic the effect of DNA binding by decreasing thermal stability and increasing catalytic activity. Residues that are involved in critical protein–protein or protein–DNA interactions are indicated (PARP-2 residues listed above PARP-3 residues). (**B**) Top: DNA-independent automodification activity of PARP-2 L269A mutant compared to PARP-2 WT using the colorimetric assay (60 nM protein, 25 μM NAD^+^). Bottom: thermal stability of PARP-2 WT and HD mutant L269A determined by DSF (5 μM protein). The *T*_M_ represents the average of three independent experiments. The error bars represent standard deviations. (**C**) Top: DNA-independent automodification activity of PARP-3 HD mutants using the radioactive assay with 1.5 μM PARP-3 WT or mutants and 2.5 μM NAD^+^ (2.0 μM NAD^+^: 0.5 μM NAD^+ 32^P). Bottom: relative thermal stability of PARP-3 HD mutants determined by DSF (5 μM protein). The average of three independent experiments are shown with associated standard deviations. (**D**) Thermal stability of PARP-2 and PARP-3 WT or mutants in the presence of DNA (5 μM protein, 2.5 μM DNA). The Δ*T*_M_ represents the difference between the average *T*_M_ obtained in the absence of DNA (three replicates with associated standard deviations) and the average *T*_M_ obtained in the presence of DNA (three replicates with associated standard deviations) (PARP-2: 28-bp duplex 5′P; PARP-3: 47-bp duplex with 5′P nick).

### Radioactive PARP automodification assay

The radioactive PARP automodification assay was performed using 1.5 μM PARP-3 WT or mutants, 2.4 μM DNA when indicated, and 2.5 μM NAD^+^ (2.0 μM NAD^+^: 0.5 μM NAD^+ 32^P) for 1 h at room temperature (RT) in 18 mM HEPES, pH 8.0, 150 mM NaCl, 0.5 mM TCEP and 10 μg/ml BSA (bovine serum albumin). Reactions were stopped by the addition of protein loading buffer, separated on a 12% SDS-PAGE, and exposed on a phosphorimager screen.

### PARP colorimetric assay

The PARP colorimetric assay was performed essentially as described (19). 60 nM of each hexahistidine-tagged protein was incubated with 480 nM DNA and 25 μM NAD^+^ (20 μM NAD^+^:5 μM biotin-NAD^+^) for indicated times. In Figure 1C–E, PARP-1, PARP-2 and PARP-3 were incubated with DNA templates as indicated. In Figures 3B and 4D, PARP-2 WT and mutants were incubated with a 28-bp duplex containing a 5′P (Supplementary Figure S1, template 18). PARP-1 and PARP-3 WT and mutants were incubated with the dnick 5′P template (Supplementary Figure S1, template 2).

### PARP-2 western blot assay

PARP-2 WT (60 nM) was incubated with 60 nM DNA (dnick or dnick 5′P) for 30 min at RT in the presence of 25 μM NAD^+^ in the same buffer conditions as the radioactive and colorimetric assays. In Supplementary Figure S6, PARP-2 WT and L269A (60 nM) were incubated with 25 μM NAD^+^ in the absence of DNA. Reactions were stopped by the addition of SDS-PAGE loading buffer, incubated for 5 min at 95°C, resolved on 10% SDS-PAGE (50 ng of protein) and blotted onto Hybond-ECL Nitrocellulose membrane (Bio-Rad). Membranes were blocked for 1 h (RT) in the following buffer: tris-buffered saline with tween (TBST; 20 mM Tris pH 7.5, 150 mM NaCl, 0.1% Tween 20) supplemented with 5% blocking-grade blocker (Bio-Rad). Blots were incubated 1 h (RT) with a 1:2000 anti-PAR antibody (Trevigen) in blocking buffer and washed with TBST and TBS then incubated with 1:7000 HRP conjugated donkey anti-rabbit antibody (Santa Cruz Biotechnology) in 1% blocking-grade buffer in TBST. Blots were washed and developed with SuperSignal West Pico Chemiluminescent Substrate (Thermo Scientific).

### Fluorescence polarization DNA binding assay

PARP-1 and PARP-2 fluorescence polarization experiments were performed using a 28-bp duplex carrying a 5′P on the terminus of one strand (Supplementary Figure S1, template 18) and a 5′ fluorescein derivative (6-FAM) on the terminus of the other strand. PARP-3 assays were performed using a 47-bp duplex containing a 5′ phosphorylated nick at position 24 (Supplementary Figure S1, template 8) and a 5′ 6-FAM group on the terminus of one strand. PARP-1 and PARP-2 reactions were performed essentially as described (19). PARP-3 reactions were performed as above in a lower ionic strength buffer (12 mM HEPES pH 8.0, 30 mM KCl, 0.12 mM EDTA, 5.5 μM β-mercaptoethanol, 0.05 mg/ml BSA and 4% glycerol), intermediate ionic strength buffer (12 mM HEPES pH 8.0, 60 mM KCl, 0.12 mM EDTA, 5.5 μM β-mercaptoethanol, 0.05 mg/ml BSA and 4% glycerol) and regular ionic strength buffer as described previously (19). Each binding curve is a representative experiment. The reported *K*_D_ represents an average of three independent experiments.

### Fluorescence polarization release assay

The fluorescence polarization NAD^+^ dependent release assay was performed essentially as described (30) with 1 mM NAD^+^. The PARP-1 assay was performed using 200 nM protein and 100 nM DNA (28-bp duplex 5′P, 5 nM fluorescently labeled). The PARP-2 assay was performed using 2.5 μM protein and 1.25 μM DNA (28-bp duplex 5′P, 5 nM fluorescently labeled). PARP-3 was performed using 4.0 μM protein and 1 μM DNA (47-bp duplex with 5′P nick, 5 nM fluorescently labeled) in the low ionic strength buffer described above.

### Differential scanning fluorimetry

Differential scanning fluorimetry experiments were performed essentially as described (20) using 5 μM protein and 2.5 μM DNA when indicated. Fluorescence emission was measured as the temperature was increased from 20 to 85°C on a Roche LightCycler 480 RT-PCR. In Figure 5C, the melting temperature in the absence of DNA was subtracted from the *T*_M_ in the presence of a 28-bp duplex 5′P (PARP-2) or a 47-bp duplex with 5′P nick (PARP-3). Reactions for PARP-3 were conducted at lower ionic strength (25 mM HEPES pH 8.0, 50 mM NaCl, 1 mM EDTA and 0.1 mM TCEP). The Δ*T*_M_ values shown represent an experiment performed in triplicate. A Boltzmann sigmoid was fit to the data to determine *T*_M_ values (KaleidaGraph).

## RESULTS

### PARP-2 and PARP-3 are selectively activated by 5′ phosphorylated DNA breaks

PARP-3 was only recently confirmed to be a DNA-dependent PARP (6,9). Consistent with its role in DNA DSB repair, PARP-3 is activated by DNA containing DSBs *in vitro* (6). The ability of PARP-3 to be activated by DNA SSBs has not been reported; therefore, we tested PARP-3 activity in the presence of a DNA dumbbell substrate containing a single, centrally placed nick (dnick). This DNA structure models an SSB as well as intermediates formed during the process of DSB repair. The dnick template activated PARP-3 modestly; however, the simple addition of a 5′ phosphate group to this template (dnick 5′P) led to a substantial increase in the level of PARP-3 activation in an automodification assay using radiolabeled NAD^+^ (Figure 1B).

To further investigate the importance of a 5′P for PARP-3 activation, we measured catalytic activity on a panel of DNA templates (nicks, gaps, blunt ends, 5′ or 3′ extensions) with or without a 5′P using a colorimetric assay (Figure 1C; see also Supplementary Figures S1 and S2). The presence of a 5′P stimulated PARP-3 activity when compared to identical templates without a 5′P, and PARP-3 was most efficiently activated by nick-containing templates harboring a 5′P (Figure 1C, templates 2 and 8). Templates containing blunt ends with a 5′P also activated PARP-3 (templates 12 and 18), but less so than 5′ phosphorylated nicks. Interestingly, the introduction of a one-nucleotide gap, rather than a nick, in the 5′ phosphorylated templates led to a substantial decrease in PARP-3 activation (Figure 1C; template 2 versus template 5 and template 8 versus template 10). Similarly, the addition of two-nucleotide extensions to the 3′ and 5′ ends of the DNA reduced DNA-dependent activation compared to the blunt-ended template (Figure 1C; templates 14 and 16 versus template 12). As a control for structural specificity, switching the phosphate to the 3′ terminus of a break resulted in a large reduction in DNA-dependent activation relative to the 5′P (Figure 1C, templates 3 and 19).

Using the same panel of DNAs and reaction conditions, we evaluated whether the same selective activation by 5′ phosphorylated DNAs existed for PARP-1 and PARP-2. PARP-2 also showed a pattern of preferential activation by DNA templates with a 5′P (Figure 1D; see also Supplementary Figures S2 and S3). For PARP-2, 5′P nicks and 5′P blunt-ended templates were equally the most potent activators (templates 2, 8, 12 and 18). Similar to PARP-3, the activation of PARP-2 was greatly reduced by the replacement of the 5′P by a 3′P (Figure 1D; template 2 versus template 3 and template 18 versus template 19) and the addition of two-nucleotide extensions at the end of blunt DNA (Figure 1D; templates 14 and 16 versus template 12). Changing the nick site by the addition of a one-nucleotide gap also decreased the ability of the DNA to activate PARP-2 (Figure 1D; template 2 versus template 5, and template 8 versus template 10).

In contrast to PARP-2 and PARP-3, the preferential activation by 5′ phosphorylated breaks was not observed for PARP-1 (Figure 1E; see also Supplementary Figure S2). Indeed, all templates activated PARP-1 to a similar level, except for the single-stranded DNA template (poly dT), which also did not activate PARP-2 or PARP-3. Importantly, PARP-1 was activated regardless of the phosphorylation status of the DNA ends. The robust and preferential activation of PARP-2 and PARP-3 by 5′ phosphorylated breaks, and in particular 5′ phosphorylated nicks, suggests that PARP-2 and PARP-3 activation is mediated by specific DNA damage intermediates and implies a role at particular steps of the repair process.

### PARP-2 and PARP-3 release from DNA upon production of PAR

The low activity levels of PARP-2 and PARP-3 in the presence of unphosphorylated DNA had been prohibitive in our biochemical studies of PARP-2 and PARP-3 mechanism of action and regulation. The robust activity in the presence of 5′ phosphorylated breaks is likely to be more physiologically relevant and has allowed us to address key biochemical questions. PARP-1 releases from DNA upon automodification induced by the presence of NAD^+^ (30,31) (Figure 2, left panel), and it has been proposed that the progressive accumulation of negatively charged PAR induces this release mechanism. PARP-3 has been shown to make relatively short polymers of up to 15 units (6), in contrast to PARP-1 and PARP-2, which make longer polymers of up to 200 units (1). Therefore, we have tested the ability of PARP-2 and 3 to release from a 5′P DNA (Figure 2, middle and right panels). In this assay, the decrease in polarization of a fluorescently labeled probe is measured over time as the PARP protein is released from the DNA upon automodification triggered by the addition of NAD^+^. Despite the smaller chains of PAR, PARP-3 was released from a 5′ phosphorylated DNA template upon addition of NAD^+^ (Figure 2, right panel). Thus, the short size of the polymer does not prevent PARP-3 from releasing from DNA. We also observed a rapid release of PARP-2 from 5′P DNA (Figure 2, middle panel) indicating that each of the DNA-dependent PARPs release from DNA upon automodification.

### The N-terminal regions (NTRs) of PARP-2 and PARP-3 are not strictly required for DNA-dependent activation

The NTRs of PARP-2 and PARP-3 show no sequence conservation with PARP-1 and have not been structurally or functionally characterized. Therefore, we investigated the NTR contribution to DNA binding affinity and catalytic activation of PARP-2 and PARP-3, and compared this with data for PARP-1. NTR deletion mutants were created such that only the WGR and CAT domains were expressed: ΔNTR-PARP-2 deleted residues 1–70 and ΔNTR-PARP-3 deleted residues 1–40. ΔNTR-PARP-2 showed a ∼4-fold reduction in DNA binding affinity compared to WT PARP-2 (Figure 3A). ΔNTR-PARP-3 exhibited a ∼3.5-fold decrease in DNA binding affinity compared to WT PARP-3. The binding deficiencies of these NTR deletions are modest in comparison with the NTR deletion of PARP-1 (deletion of residues 1–517), which decreases DNA binding affinity 1000-fold, from ∼10 nM for PARP-1 full-length (FL) to >40 μM for ΔNTR-PARP-1 (30). The NTR of PARP-1 is composed of several domains that contribute to DNA binding affinity and activation. Thus, in contrast to PARP-1, the NTRs of PARP-2 and PARP-3 appear to make only partial contributions to the overall binding affinity to DNA breaks.

We next tested the DNA-dependent activation of ΔNTR-PARP-2 and ΔNTR-PARP-3 (Figure 3B). In PARP-1, the NTR contains the Zn1 and the Zn3 domains that are essential for DNA-dependent activation. Indeed, point mutations in these domains abolish DNA-dependent activity, without affecting DNA binding ability, due to disruption of the communication between the regulatory domains (Zn1, Zn3, WGR) and the CAT (20,30). Thus, deletion of PARP-1 NTR leads to an enzyme that only retains DNA-independent activity (Figure 3B). In contrast, ΔNTR-PARP-2 and ΔNTR-PARP-3 retain a measurable level of DNA-dependent activity, albeit reduced when compared to the WT enzymes (Figure 3B), indicating that the NTRs of PARP-2 and PARP-3 are not essential for DNA-dependent activity. Thus, the NTRs of PARP-2 and PARP-3 do not perform the same functions as the NTR of PARP-1 in DNA binding and DNA-dependent activation. We therefore investigated the WGR as the chief DNA-dependent regulatory domain of PARP-2 and PARP-3.

### PARP-2 and PARP-3 are primarily regulated through the WGR domain

Recent structural and biochemical analysis of PARP-1 have highlighted a central role for the WGR domain in allosterically regulating the CAT domain in collaboration with the Zn1 and Zn3 domains (20,30). We performed structure-guided mutagenesis of the WGR domain of PARP-2 and PARP-3 to understand the WGR contribution to DNA binding and DNA-dependent activation. An NMR structure of the PARP-3 WGR domain (PDB code: 2EOC) and a crystal structure of the PARP-3 CAT domain (PDB code: 3C49) were aligned to the PARP-1/DNA crystal structure (PDB code: 4DQY), which contains a complex of these domains bound to DNA and the regulatory domains, Zn1 and Zn3 (Figure 4A). In the superimposed model, PARP-3 WGR is positioned to interact with the DNA break near the 5′ terminus in a manner similar to PARP-1. In the PARP-1/DNA crystal structure, residue W589 makes contact with the deoxyribose sugars of nucleotides located at the 5′ end of the break (Figure 4A), and its mutation to an alanine (W589A) greatly reduces PARP-1 DNA-dependent activity (20). We have mutated the equivalent residues in PARP-2 (W138A) and PARP-3 (W101A) and have tested their ability to bind to DNA (Figure 4B and C, Supplementary Figure S4) and their ability to be activated by DNA (Figure 4D). While PARP-2 W138A shows a reduction in affinity to ∼28% of WT, PARP-3 W101A binds to DNA similar to WT (Figure 4C, Supplementary Figure S4). Similar to PARP-1 W589A, both PARP-2 W138A and PARP-3 W101A show a complete loss of DNA-dependent activity (Figure 4D), indicating that the Trp contact with DNA is essential to activation.

In PARP-1, WGR residue R591 performs a crucial role in DNA-dependent activation by interacting with both Zn1 and the HD, creating a bridge between the DNA-binding interface and the CAT (Figure 4A). Mutation R591A does not affect PARP-1 affinity for DNA (30), but eliminates DNA-dependent activity (20). Since PARP-2 and PARP-3 do not have a Zn1 domain, the corresponding arginines in PARP-2 (R140) and PARP-3 (R103) (Figure 4B) are expected to perform different functions, or perhaps to have no critical importance. Mutation of these arginine residues in PARP-2 (R140A) and PARP-3 (R103A) reduced affinity for DNA to ∼25% of WT (Figure 4C, Supplementary Figure S4) and entirely abolished DNA-dependent catalytic activity (Figure 4D), without affecting DNA-independent activity (Supplementary Figure S5). Based on the structural model, R140 and R103 could potentially make direct contributions to the DNA binding interface, in contrast to the domain-domain interface seen with PARP-1.

We also mutated residues PARP-2 Y188 and PARP-3 Y150. These residues are expected to approach the DNA end bearing the 5′ phosphate, a structural feature shown in a previous section to selectively stimulate PARP-2 and PARP-3 (Figure 4A). PARP-1 has a phenylalanine at the equivalent position (F638) (Figure 4B). Mutating the Tyr to a Phe to mimic PARP-1 (PARP-2 Y188F and PARP-3 Y150F) yielded mutants with no DNA-dependent activity (Figure 4D). In PARP-2, mutant Y188F showed a substantial decrease in affinity for DNA (∼7% of WT), while PARP-3 Y150F showed a more modest reduction in affinity (∼54% of WT). Therefore, PARP-2 residue Y188 seems to make an important contribution to DNA binding and to activation. PARP-1 residue F638 is located on a loop that is involved in contacts with Zn3 and the HD. Our results suggest that without a Zn3 domain, PARP-2 and PARP-3 use this loop of the WGR to play a direct role in DNA binding and activation, thus replacing the Zn3 contribution to the allosteric activation mechanism.

PARP-1 residue N567 is located on the WGR surface opposite to the DNA binding interface, where it contacts the HD near α-helix E (Figure 4A). N567 is essential for DNA-dependent activity (20). Mutation of the equivalent asparagine residues in PARP-2 (N116A) and PARP-3 (N80A) entirely abolished DNA-dependent activity, with mild (∼45% of WT for PARP-2) to no deficiency (PARP-3) in DNA binding (Figure 4C and D). Thus, the results suggest that similar to PARP-1, important communication is mediated between the WGR domain and the HD of PARP-2 and PARP-3 that is essential to support DNA-dependent activity. All PARP-2 and PARP-3 WGR mutants retained DNA-independent activity similar to WT as expected (Supplementary Figure S5).

Collectively, our mutagenesis data indicate that the WGR domain of PARP-2 and PARP-3 is essential for DNA-dependent activity, forming contacts with both the DNA and the catalytic domain. Based on homology to PARP-1 and the mutagenesis results, we expect that PARP-2 and PARP-3 will interface with DNA and the HD in a similar way. However, the WGR domain of PARP-2 and PARP-3 seems to make a greater contribution to DNA damage-dependent activity compared to PARP-1, which is more dependent on the domains located in its NTR. Thus, the WGR domains of PARP-2 and PARP-3 likely perform some of the functions attributed to the domains contained in the NTR of PARP-1, in particular, contributing to the stabilization of the activated conformation of the catalytic domain.

### A conserved allosteric activation mechanism for PARP-1, PARP-2 and PARP-3

The crystal structure of PARP-1 in complex with DNA damage and supporting biochemical data indicated that DNA damage-dependent activation of PARP-1 results from a destabilizing structural transition in the CAT domain that is imparted through a series of key interdomain contacts (20,24) (Figure 5A). Specifically, the crystal structure indicated that the hydrophobic core of the HD was distorted when compared to the HD in crystal structures of individual CAT domains in the absence of regulatory domains and DNA. Mutations that directly disrupt the hydrophobic core of the HD mimicked the effect of DNA binding, stimulating PARP-1 activity in the absence of DNA. These activating mutations destabilized the CAT and led to a striking anti-correlation between DNA-independent activity and thermal stability, with lower thermal stability corresponding to higher catalytic activity. Furthermore, a decrease in thermal stability of WT PARP-1 was observed in the presence of DNA (20), consistent with a DNA-dependent destabilization mechanism.

The WGR mutagenesis shown in the previous section indicated that PARP-2 and PARP-3 mediate a network of communication between the WGR and CAT domains that is similar to that of PARP-1. To determine whether PARP-2 and PARP-3 also have a DNA-dependent mechanism of activation that relies on HD distortion, we targeted the HD hydrophobic core of PARP-2 and PARP-3 with mutagenesis. We chose HD residues that when mutated in PARP-1 showed the greatest increase in DNA-independent activity, and correspondingly a lower thermal stability as measured by differential scanning fluorimetry (DSF) (20). Consistent with the HD destabilization mechanism, PARP-2 mutant L269A (corresponding to PARP-1 L713A) showed a marked increase in DNA-independent activity relative to PARP-2 WT over a time course measured by the colorimetric assay (Figure 5B, top panel: 8.9 ± 1.1-fold increase at the 15 min time point)(see also Supplementary Figure S6). Furthermore, PARP-2 mutant L269A showed a substantial decrease in thermal stability relative to WT (Figure 5B, bottom panel). PARP-3 mutants L233A and L286A (corresponding to PARP-1 L713A and L765A) also showed an increase in DNA-independent activity when tested in an activity assay using radiolabeled NAD^+^ (Figure 5C, top panel). These mutations also showed a reduction of thermal stability relative to WT (Figure 5C, bottom panel).

We also investigated whether PARP-2 and PARP-3 were destabilized in the presence of DNA, as previously reported for PARP-1 (20). Indeed, PARP-2 showed a reduction in thermal stability in the presence of an activating DNA (Figure 5D), consistent with the destabilization mechanism. Strikingly, only DNA carrying a 5′P showed a decrease in thermal stability, while non-phosphorylated DNA did not elicit a change in thermal stability. Thus, PARP-2 uses a destabilization mechanism for activation, and the specific DNA structures that increase PARP-2 catalytic activity (as seen in Figure 1C) are required for the observed change in thermal stability (Figure 5D). Importantly, the PARP-2 mutant N116A does not support DNA-dependent activity (Figure 4D), and correspondingly did not show a decrease in thermal stability in the presence of 5′ phosphorylated or non-phosphorylated DNA (Figure 5D, left), as expected for a mutant that disrupts communication between the WGR and HD domains (Figure 5A). As a control, the PARP-2 catalytic active site mutant E545A was destabilized similar to WT, consistent with its ability to form WGR-HD contacts and thereby lower thermal stability, even though it is catalytically inactive (Figure 5D and Supplementary Figure S7).

Ligand binding typically contributes to an increase in the thermal stability of a protein. Destabilization of the HD makes opposing contributions that lower thermal stability; however, the stabilizing forces of ligand binding are still relevant to interaction with DNA. Indeed, when the destabilization mechanism of PARP-2 was inactivated with mutation N116A, an increase in thermal stability was observed with both 5′ phosphorylated and non-phosphorylated DNA templates (Figure 5D). The increase in thermal stability arises from contacts formed upon binding to DNA, and the N116A mutation prevents the counteracting destabilization. Increases in thermal stability were previously observed for PARP-1 mutants D45A and W318R, which have robust interaction with DNA but are unable to form key interdomain contacts and thus impart HD destabilization (20).

We observed an overall increase in PARP-3 thermal stability in the presence of DNA (Figure 5D, right), which reflects that the stabilizing forces of PARP-3 interaction with DNA are the major contributors to overall thermal stability. PARP-3 thermal stability in the absence of DNA is relatively low (*T*_M_ ∼ 31°C) compared to PARP-1 (*T*_M_ ∼44°C) and PARP-2 (*T*_M_ ∼41°C); therefore, we anticipate that PARP-3 benefits more in terms of thermal stability from binding to DNA than PARP-1 and PARP-2, overriding the contributions of HD destabilization. Importantly, however, the stabilizing effect of DNA was strongly diminished in the presence of the 5′ phosphorylated DNA compared to the non-phosphorylated DNA (Figure 5D). We interpret this reduction in stability in the presence of 5′ phosphorylated DNA to reflect the HD destabilization mechanism, consistent with the increase in catalytic activity observed on DNA bearing a 5′P (Figure 1C). Consistent with this interpretation, PARP-3 mutant N80A, which disrupts the WGR-HD interaction and abolishes DNA-dependent activity but not DNA binding affinity (Figure 4C), showed the same level of thermal stability on both 5′ phosphorylated and non-phosphorylated templates (Figure 5D). Furthermore, the ΔNTR mutation of PARP-3, which retains DNA-dependent activity (Figure 3B), also showed a decrease in stabilization in the presence of the 5′ phosphorylated template similar to WT (Figure 5D). Interestingly, PARP-3 mutant Y150F was less efficient at responding to the 5′ phosphorylated template, as expected given its lack of DNA-dependent activity. Y150 is modeled in close proximity to the 5′ DNA end and could therefore be important for coupling 5′P detection to catalytic activation. Together, our results indicate that in response to binding DNA damage, PARP-2 and PARP-3 share with PARP-1, a common mechanism of activation that involves WGR contacts that destabilize the HD and lead to an increase in catalytic activity (Figure 5A).

Our model suggests that DNA breaks bearing a 5′P specifically activate PARP-2 and PARP-3 through a more efficient execution of the HD destabilization mechanism. However, we wanted to confirm that the differences in activity observed could not be explained by a significant increase in binding affinity toward 5′P DNA breaks. As expected, PARP-1 showed affinities in the low nanomolar range for both 5′ phosphorylated and non-phosphorylated templates (Supplementary Figure S8). PARP-3 showed no difference in binding affinity for the 5′ phosphorylated versus the non-phosphorylated DNA (Supplementary Figures S8 and S9). PARP-2 exhibited an ∼2.5-fold increase in affinity for DNA with a 5′ phosphorylated end over the non-phosphorylated DNA (Supplementary Figure S8). This result indicates that PARP-2 DNA binding affinity is partially determined by the phosphorylation status of the DNA end, which could explain the notable reduction in binding affinity for mutant Y188F, which is positioned near the 5′ DNA end. However, we expect that the 2.5-fold increase in affinity on a 5′P DNA end is unlikely to explain the ∼15-fold difference in DNA-dependent activation observed between 5′ phosphorylated and non-phosphorylated templates (Figure 1C; compare template 11 to template 12 and template 17 to template 18). Furthermore, the thermal stability experiments that monitor activation were performed at protein concentrations at which both DNAs are bound at similar levels, and only the 5′P DNA was able to reduce thermal stability.

## DISCUSSION

Our analysis indicates that PARPs 1, 2 and 3 have a similar mechanism of activation in response to binding to DNA damage. DNA damage-dependent activation destabilizes the structure of the HD in a way that leads to an increase in the efficiency of PAR production. We anticipate that the WGR-HD interface will be fairly similar among PARPs 1, 2 and 3, based on the observation that HD destabilization is lost when a conserved WGR-CAT interface residue is mutated (N116 in PARP-2, N80 in PARP-3, N567 in PARP-1). Thus, the allosteric communication between WGR and CAT is likely to operate in a similar manner among all DNA damage-dependent PARPs. By contrast, the details of the WGR interface with DNA are likely to be somewhat different among these because PARP-2 and PARP-3 lack the regulatory zinc fingers present in PARP-1, which make essential contributions to the DNA binding interface and the activation mechanism. Rather, the WGR domain is the centerpiece of DNA damage-dependent regulation for PARP-2 and PARP-3. Detailed structural analysis will be required to understand how PARP-2 and PARP-3 use their WGR domains to contact DNA and couple this interaction to contacts with the HD.

An interesting finding of our study is that the NTRs of PARP-2 and PARP-3 are not strictly required for DNA damage-dependent activation, further supporting that the WGR domain is the key regulatory element. The decrease in DNA-dependent activation observed in the NTR deletions of PARP-2 and PARP-3 could be due to their lower affinity for DNA compared to WT (Figure 3A), which could lower the efficiency of the DNA damage-dependent activation mechanism by lowering the residence time on DNA breaks. Another possibility is that the NTRs of PARP-2 and PARP-3 contain residues that are targeted for automodification. Indeed, PARP-1 automodification sites have been identified in the linker region between the BRCT and the WGR domains (22,32–34). The corresponding regions in PARP-2 and PARP-3 adjacent to the WGR domain might also contain automodification sites, which are deleted in the NTR deletions of PARP-2 and PARP-3, and thus could potentially contribute to the observed decrease in PAR formation (Figure 3B). Proteomic analysis of automodification sites in PARP-2 and PARP-3 will help to differentiate these possibilities.

The biochemical observation that 5′ phosphorylated breaks selectively stimulate PARPs 2 and 3 has interesting implications for their involvement in DNA damage repair. It is notable that the binding affinities do not change appreciably for the different models of DNA damage used in this study, indicating that PARP-2 and PARP-3 recruitment to sites of DNA damage will not likely be influenced by the fine structure of the DNA break. By contrast, robust activation in the presence of phosphorylated versus non-phosphorylated breaks suggests that PARP-2 and PARP-3 will respond to the structural details of the breaks, and this could allow PARP-2 and PARP-3 to carry out their functions at specific steps of the DNA repair process.

In the recent literature, PARP-2 and PARP-3 involvement in specific DNA repair pathways has been examined. Both appear to be involved in the kinetics of repair. In particular, PARP-3 participates in the non-homologous end-joining (NHEJ) pathway that repairs DSBs (6,9,35,36). PARP-3 co-localizes with γH2AX to sites of laser irradiation in cells (9), and its depletion causes a delay in DSB repair kinetics (6). The decrease in repair kinetics can be overcome through overexpression of XRCC4/DNA ligase IV, indicating that one role of PARP-3 at damage sites is to increase the efficiency of the final strand-joining step of repair (6). DNA ligase requires a 5′ phosphorylated, nicked DNA to carry out the ligation reaction.

**Figure 6.**
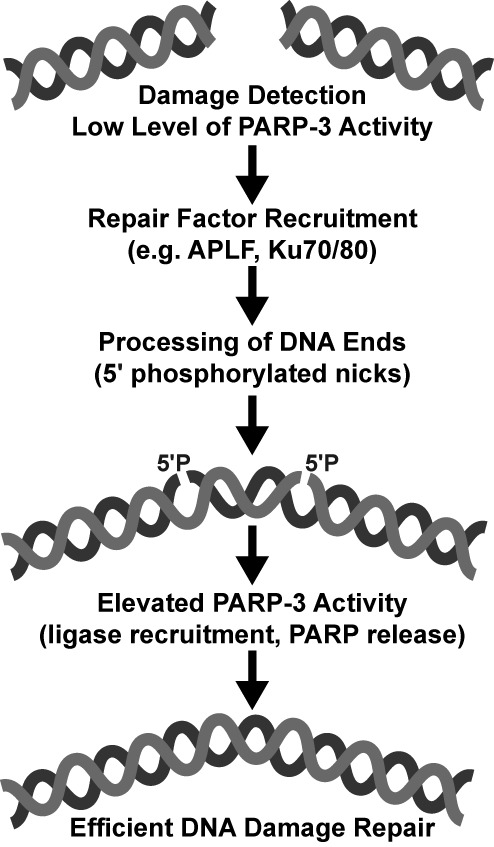
Model for PARP-3 activation during NHEJ. In the proposed model, a low level of PARP-3 activation after damage detection contributes to the recruitment of repair machinery. The DNA ends are processed during repair leading to the final substrate for DNA ligation, a phosphorylated nick (5′P). An elevated level of PARP-3 activation in the presence of the phosphorylated break could then act to recruit the ligase complex for the end-joining reaction, or could release PARP-3 so that DNA ligase can access the break. Based on our biochemical analysis, PARP-2 could function similarly in other repair pathways.

Thus, we propose that at the end of the NHEJ pathway, the presence of 5′ phosphorylated nicks in the DNA would lead to an elevated level of PARP-3 activation that could signal the presence of unsealed breaks, and the enhanced PAR formation would assist the recruitment of the ligase complex to the breaks for end-joining reaction (Figure 6). It is noteworthy that PARP-3 was most active on a nicked DNA template, whereas a gap in the broken DNA strand was far less capable of stimulating activity, further implicating PARP-3 activation in response to a specific DNA repair intermediate associated with DNA ligation.

The proposed model does not preclude PARP-3 involvement in earlier steps of the repair pathway (Figure 6). Indeed, PARP-3 has been found in a complex with several components of the NHEJ pathway, including Ku70/Ku80, DNA-PKcs and DNA ligase IV (37). The overall DNA binding affinity of PARP-3 is considerably lower than that of PARP-1 and PARP-2 (Supplementary Figure S9), suggesting that PARP-3 could potentially require partner proteins to efficiently recruit to cellular sites of DNA damage. The interaction of PARP-3 with Ku70/Ku80 is dependent on the presence of DNA (37), and could therefore potentially be involved in PARP-3 localization to sites of damage. In our proposed model, initial binding of PARP-3 to DNA could modestly activate PARP-3 in order to allow recruitment of subsequent factors, e.g. the PAR-binding factor APLF (6,38). Once the break is processed to contain two DNA nicks bearing a 5′P that are competent for the strand-joining reaction catalyzed by DNA ligase IV, hyperactivation of PARP-3 could assist in recruiting XRCC4/DNA ligase IV. Hyperactivation might instead invoke the PARP-3 release mechanism to allow ligase to have access to the break, since both PARP-3 and ligase bind at the 5′P DNA end (39). Additionally, the locally produced PAR could be metabolized to contribute to a high local concentration of ATP for the ligase reaction, as proposed for PARP-1 with DNA ligase I at the end of BER (40).

PARP-2 is thought to be involved in the BER/SSB repair pathway, since its deletion in mice induces a delay in DNA repair following exposure to DNA alkylating agents and ionizing radiation (11,12). PARP-2 is also known to interact with XRCC1, DNA pol ß, DNA ligase III (11), and various other DNA repair factors (35). Additionally, it was suggested that PARP-2 acts after PARP-1 in the SSB repair pathway (8). However, the details of specific PARP-2 roles in DNA repair processes are less developed. Based on our biochemical analysis, we suggest that PARP-2 plays a role similar to PARP-3 at the end of a repair process that involves 5′ phosphorylated nicks, by participating in the recruitment of repair factors that will allow the breaks to be ligated (Figure 6). The process of Okazaki fragment repair during replication requires repeated ligation events and could also be influenced by the specific activation of PARP-2.

In summary, our study has identified 5′ phosphorylated breaks as preferential DNA activators of PARP-2 and PARP-3. PARP-2 and PARP-3 recognition of 5′ phosphorylated DNA breaks is tied to an allosteric regulatory mechanism that elevates PAR synthesis activity through structural alterations in the catalytic domain. Although the general elements of the allosteric regulatory mechanism are similar for PARP-1, PARP-2 and PARP-3, our mutagenesis data highlight that there are differences in how these DNA damage-dependent PARPs engage DNA breaks. Further structural analysis will ultimately clarify these differences. Collectively, our study provides new insights into the specialization of the DNA-dependent PARPs and their specific roles in coordinating the efficiency of DNA repair.

## SUPPLEMENTARY DATA

Supplementary Data are available at NAR Online.

SUPPLEMENTARY DATA
