# Supplementary material for: PARP-2 and PARP-3 are selectively activated by 5′ phosphorylated DNA breaks through an allosteric regulatory mechanism shared with PARP-1
Source: Nucleic Acids Res. 2014 Jun 7;42(12):7762–75. doi: 10.1093/nar/gku474 (PMC4081085; doi:10.1093/nar/gku474)
Supplement: SUPPLEMENTARY DATA [file supp_42_12_7762__index.html]

PARP-2 and PARP-3 are selectively activated by 5′ phosphorylated DNA breaks through an allosteric regulatory mechanism shared with PARP-1 — SUPPLEMENTARY DATA 

# PARP-2 and PARP-3 are selectively activated by 5′ phosphorylated DNA breaks through an allosteric regulatory mechanism shared with PARP-1

## SUPPLEMENTARY DATA

**Files in this Data Supplement:**

- Supplementary Figure
