# Supplementary material for: PARP-2 and PARP-3 are selectively activated by 5′ phosphorylated DNA breaks through an allosteric regulatory mechanism shared with PARP-1
Source: Nucleic Acids Res. 2014 Jun 7;42(12):7762–75. doi: 10.1093/nar/gku474 (PMC4081085; doi:10.1093/nar/gku474)
Supplement: SUPPLEMENTARY DATA [file supp_gku474_nar-00652-h-2014-File008.doc]

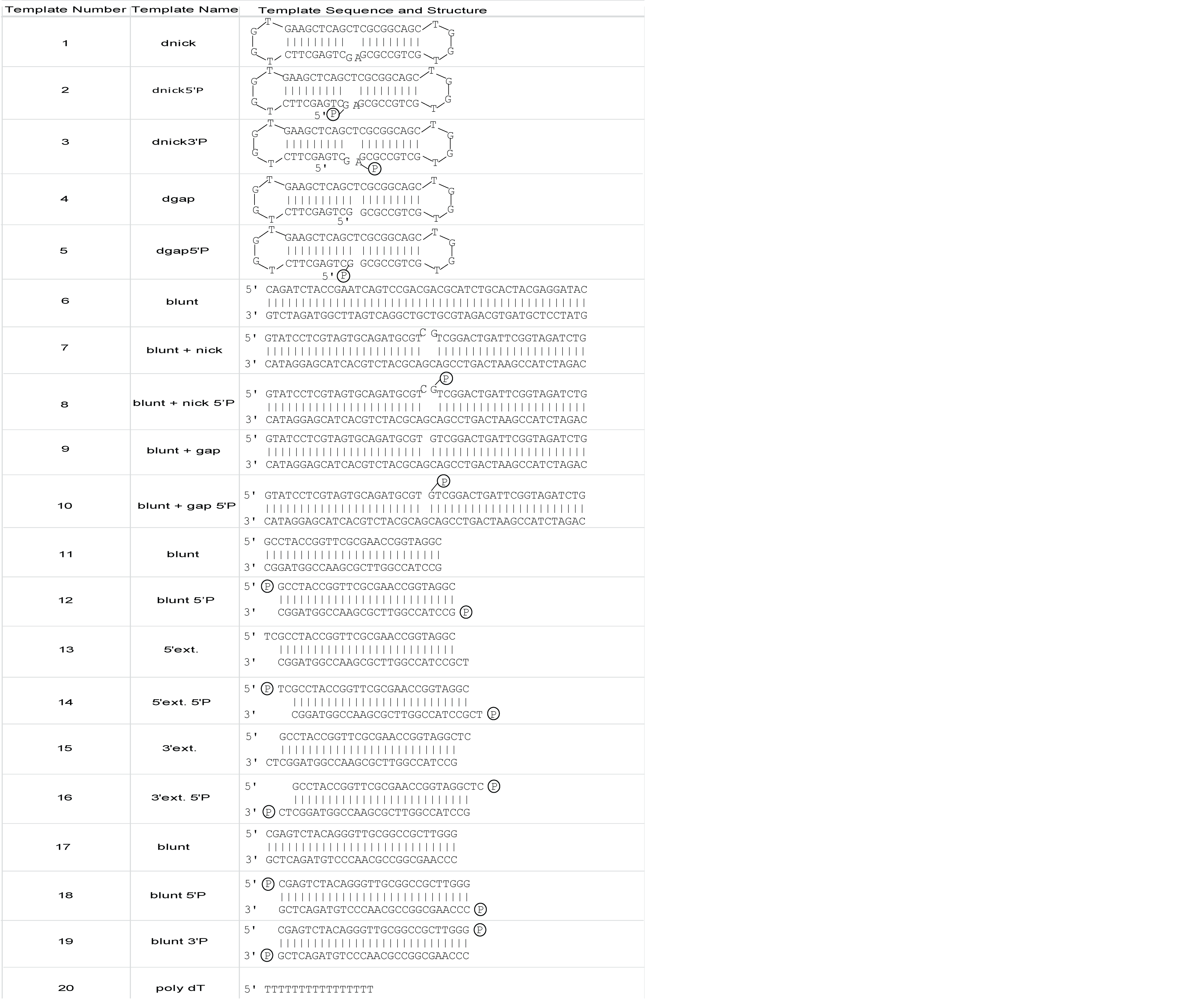


Supplementary Figure 1. Sequence and structure of the DNA templates used in this study.

Supplementary Figure 2. PARP-2 and PARP-3, but not PARP-1, show preferential activation by 5**'** phosphorylated templates. Colorimetric assay measuring PARP-1, PARP-2, and PARP-3 activity in the presence of various DNA templates at several time points using 60 nM of protein and 480 nM DNA. The numbers in parenthesis refer to the template number in Figure 1C.


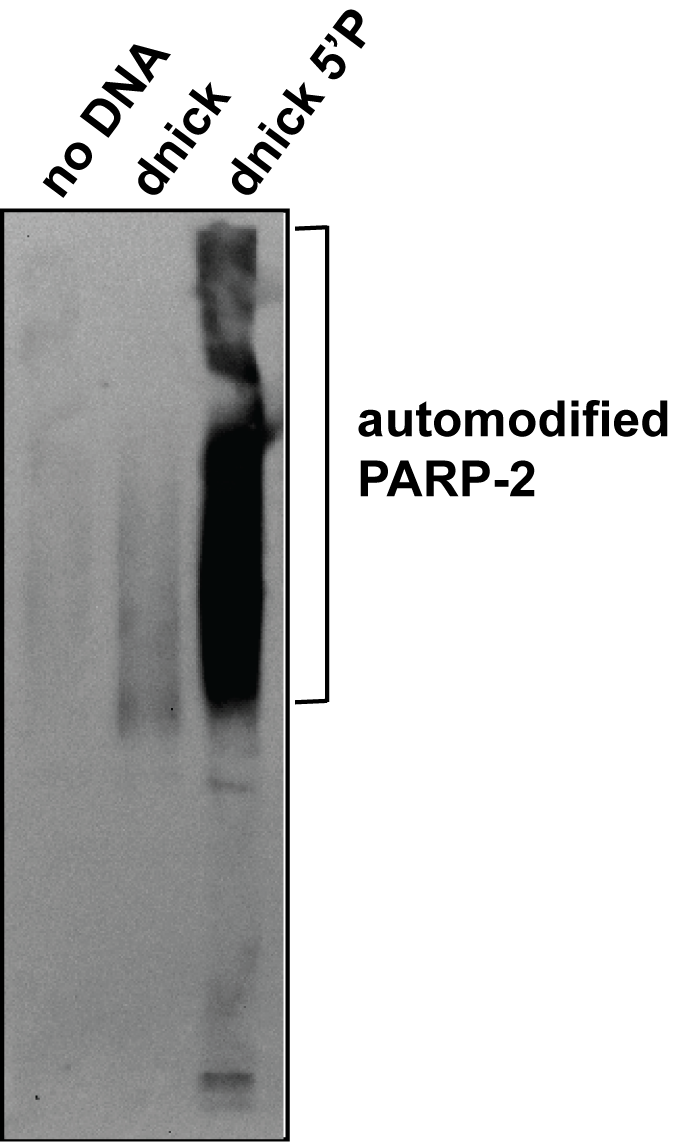


Supplementary Figure 3. Western blot assay of PARP-2 activity. PARP-2 (60 nM) was incubated with DNA (60 nM) for 30 minutes at room temperature (RT) in the presence of 25 μM NAD^+^. Reactions were resolved on SDS-PAGE and transferred to a membrane. The western blot was performed using an anti-PAR antibody (Trevigen).


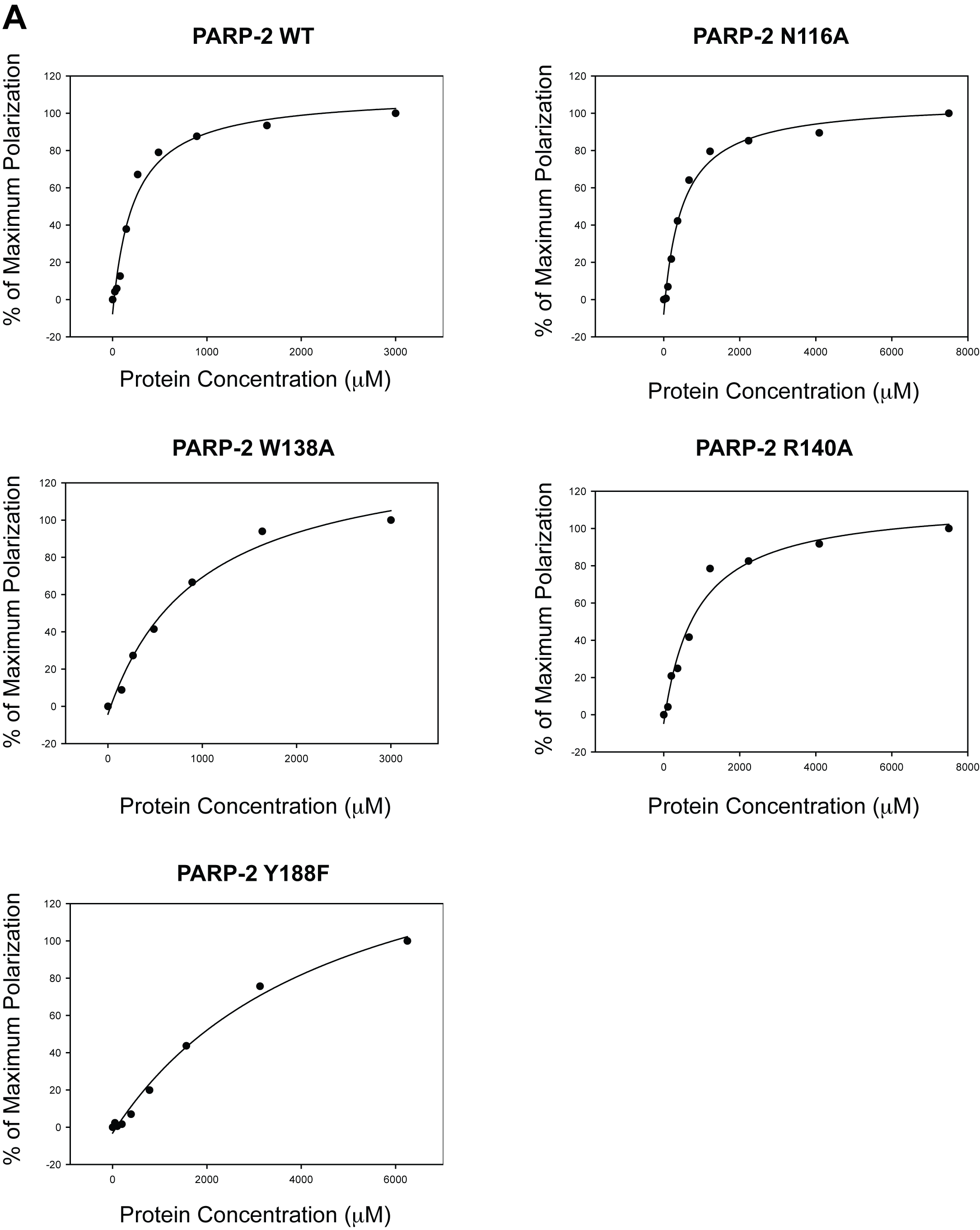

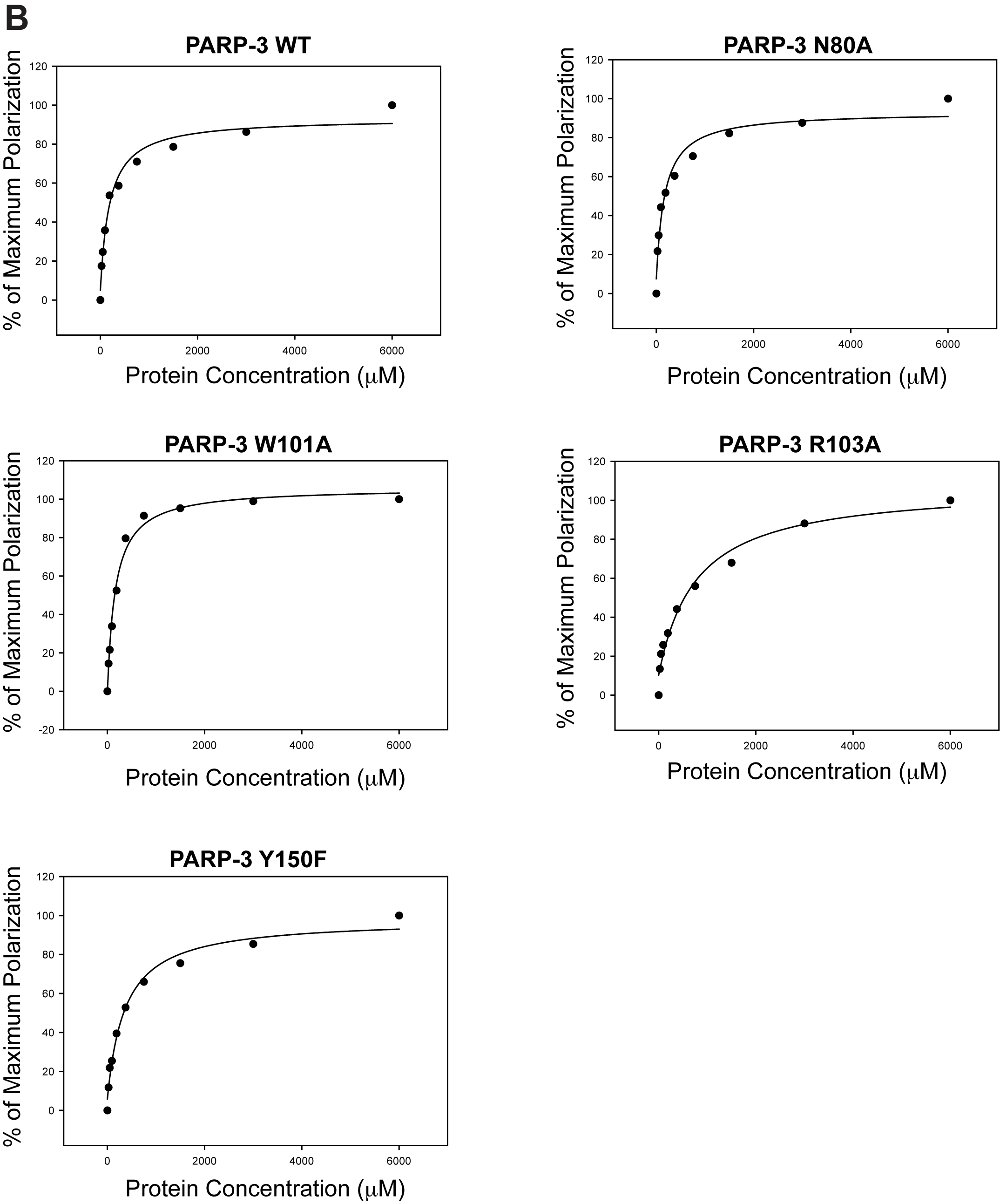


**Supplementary Figure 4. Fluorescence Polarization DNA binding experiments of PARP-2 and PARP-3 WT and mutants.** A. A fluorescently labeled 28 bp DNA duplex carrying a 5' P (template 18, 5 nM) was titrated with PARP-2 WT or mutants. B. A fluorescently labeled 47 bp DNA duplex carrying a central 5' phosphorylated nick (template 8, 5nM) was titrated with PARP-3 WT or mutants. An example of a binding assay is shown for each protein. The percentage of maximum polarization was obtained using the following formula: (F_O_-A_min_)/(A_max_-A_min_), where F_O_ is the observed polarization for a specific protein concentration, A_min_ is the polarization measured in the absence of protein, and Amax is the maximum observed polarization.


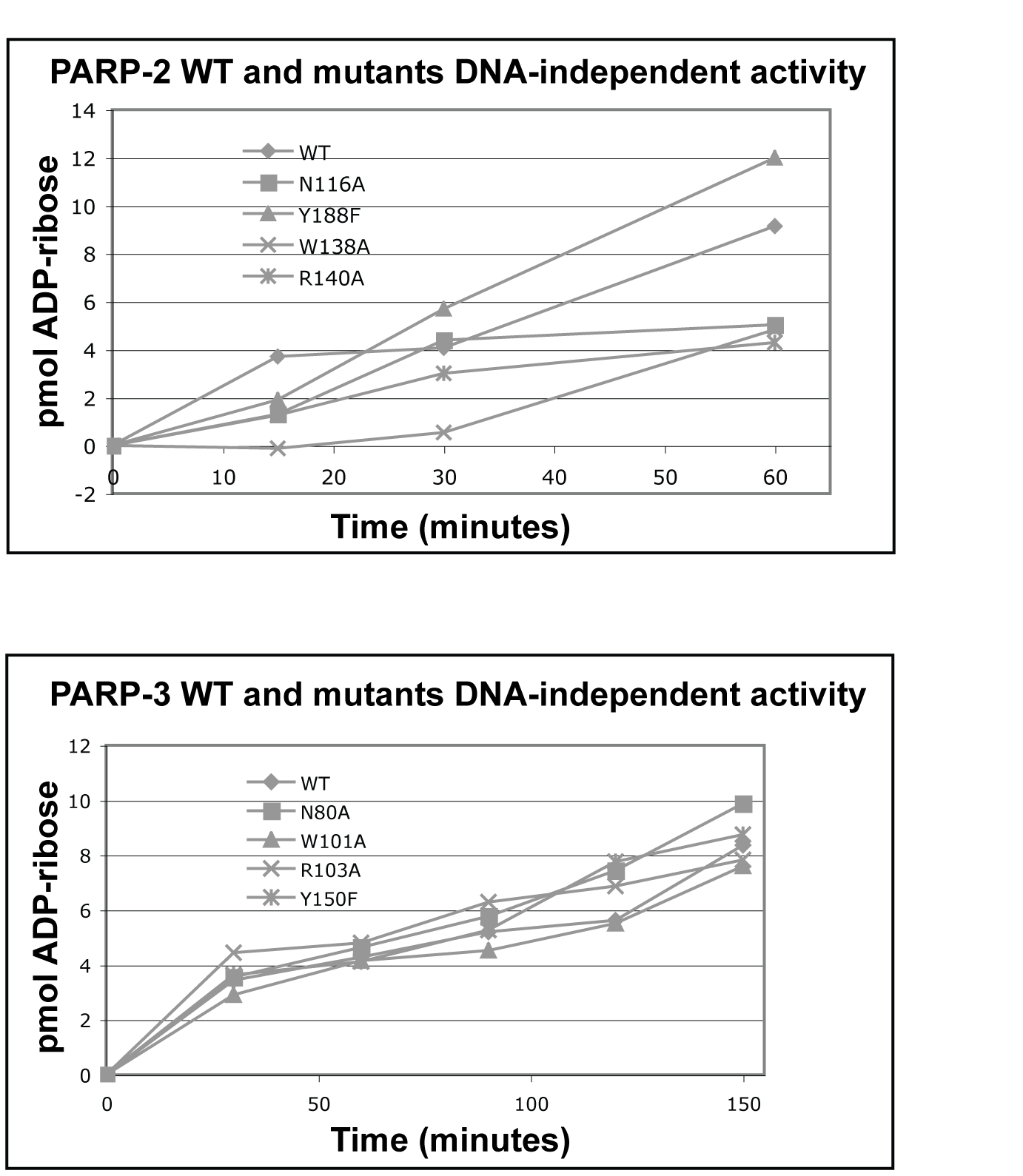


**Supplementary Figure 5. DNA-independent activity of PARP-2 and PARP-3 WGR mutants.** Colorimetric assay showing the activity of PARP-2 and PARP-3 WGR mutants (60 nM) in the absence of DNA.


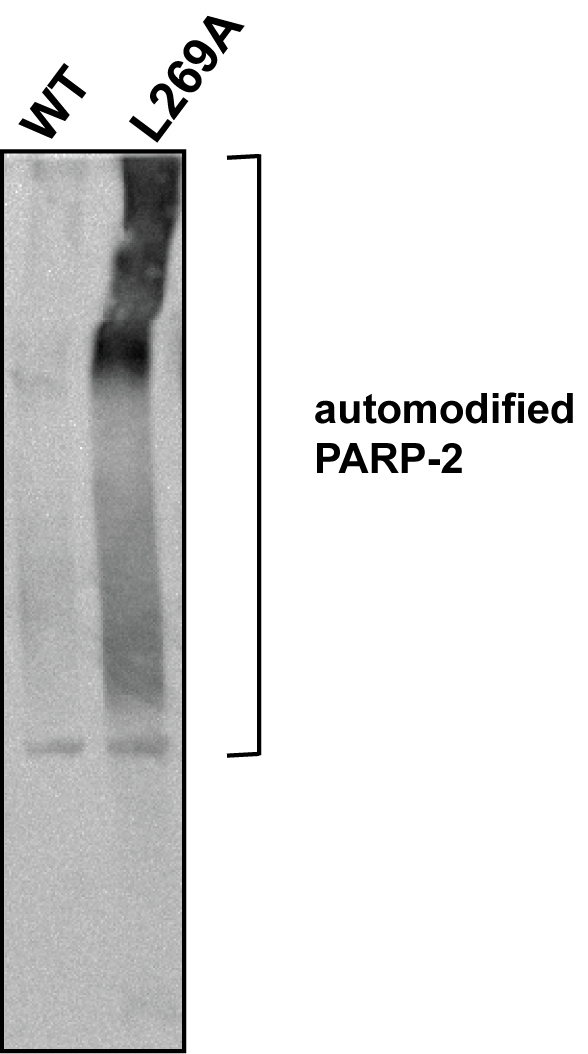


**Supplementary Figure 6.** Western blot assay comparing the DNA-independent activity of PARP-2 WT and L269A. PARP-2 WT or L269A (60 nM) was incubated with 25 µM NAD^+^ for 30 minutes at room temperature (RT). The reactions were resolved on SDS-PAGE. The western blot was performed using an anti-PAR antibody (Trevigen).


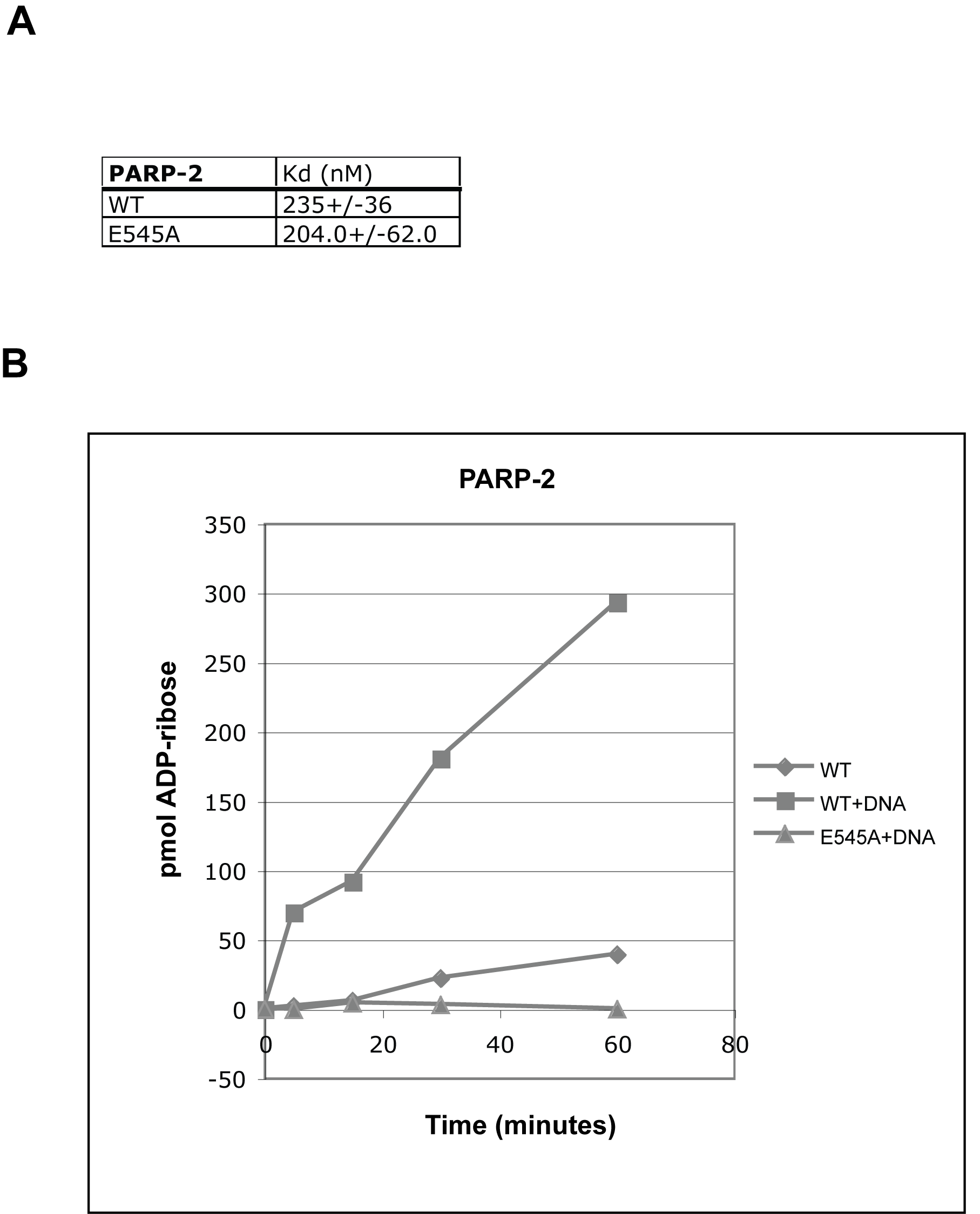


Supplementary Figure 7. The PARP-2 mutant E545A is deficient in catalytic activity but has the same affinity for DNA as PARP-2 WT. A. The binding affinities of PARP-2 WT and PARP-2 E545A were determined by fluorescence polarization. The values shown are the average K_D_ calculated from three independent experiments with the associated standard deviation. B. Colorimetric assay showing the activity of PARP-2 WT and PARP-2 E545A. The assay was performed as in Figure 4C.


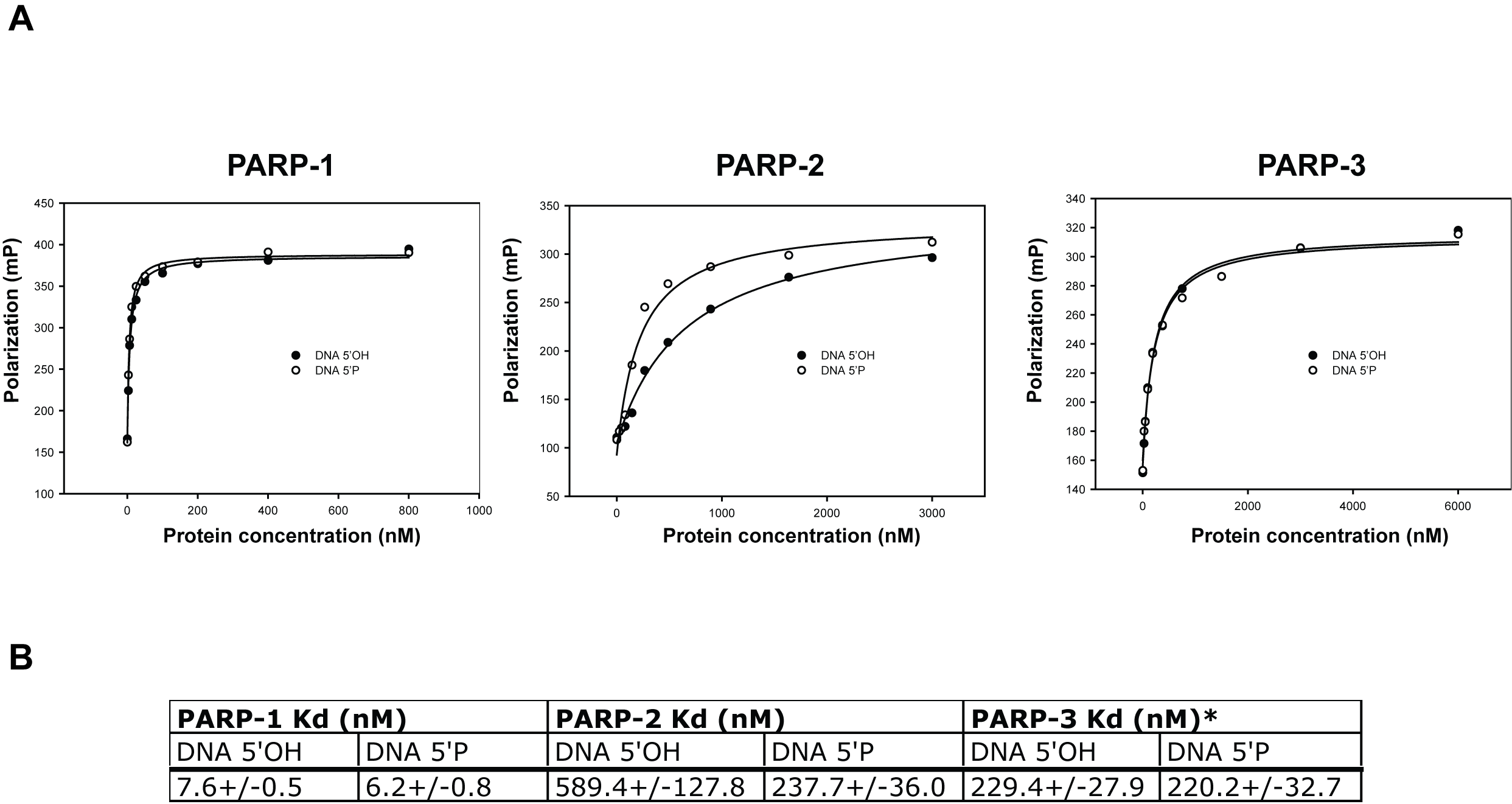


Supplementary Figure 8. PARP-2 and PARP-3 have similar affinities for 5' phosphorylated or non-phosphorylated DNA breaks. A. Fluorescence polarization DNA binding experiment. Fluorescently labeled DNA probes (5 nM) containing either a 5' P or 5' OH terminus were incubated with various concentrations of protein for 30 minutes at room temperature prior to measurement. B. The K_D_ reported represents the average derived from three independent experiments with the associated standard deviation. * The PARP-3 assay was carried out at lower salt concentration to allow DNA binding to be accurately determined (see also Supplementary Figure 5).


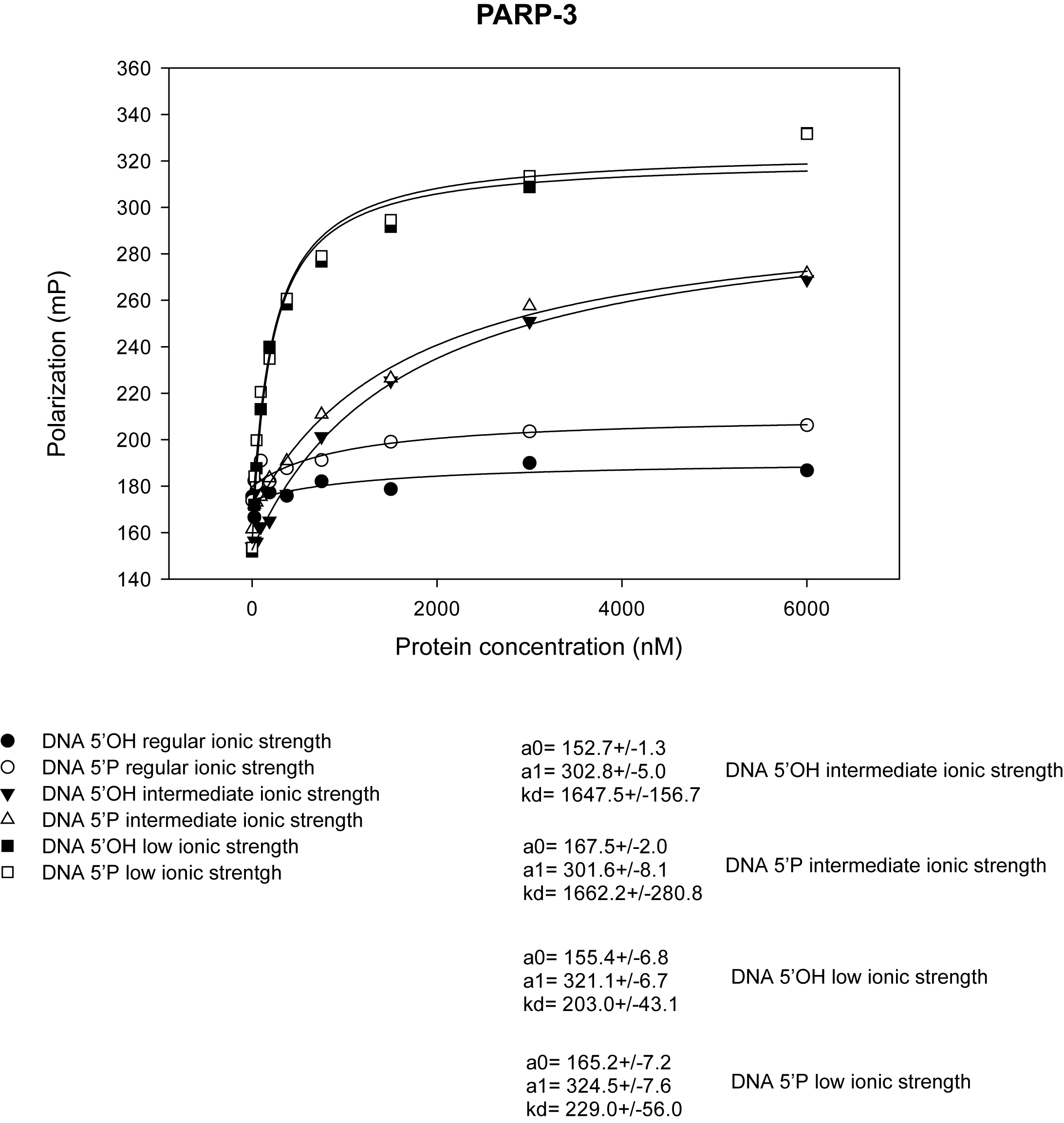


Supplementary Figure 9. PARP-3 shows no preferential binding to 5' phosphorylated DNA. Fluorescence polarization assay measuring PARP-3 binding affinity for DNA templates carrying a 5' P or 5' OH at their terminus using various ionic strength buffers. PARP-3 showed no measurable DNA binding activity in our standard ionic strength buffer. Salt concentrations were lowered in order to measure binding of PARP-3 to the DNA probes. Our data show that at various salt concentrations PARP-3 has the same affinity for both 5'phosphorylated and non-phosphorylated DNA templates. The buffer compositions are described in the Material and Methods section. The values for the K_D_, baseline polarization (ao), and maximum polarization (a1) were obtained by fitting the data to a two-state binding model.
